# Supplementary material for: Estradiol Prevents Amyloid Beta-Induced Mitochondrial Dysfunction and Neurotoxicity in Alzheimer’s Disease via AMPK-Dependent Suppression of NF-κB Signaling
Source: Int J Mol Sci. 2025 Jun 27;26(13):6203. doi: 10.3390/ijms26136203 (PMC12249544; doi:10.3390/ijms26136203)
Supplement: Supplementary file 1 [file ijms-26-06203-s001.zip › ijms-3687431-supplementary.pdf]

Supplementary Figure S1.

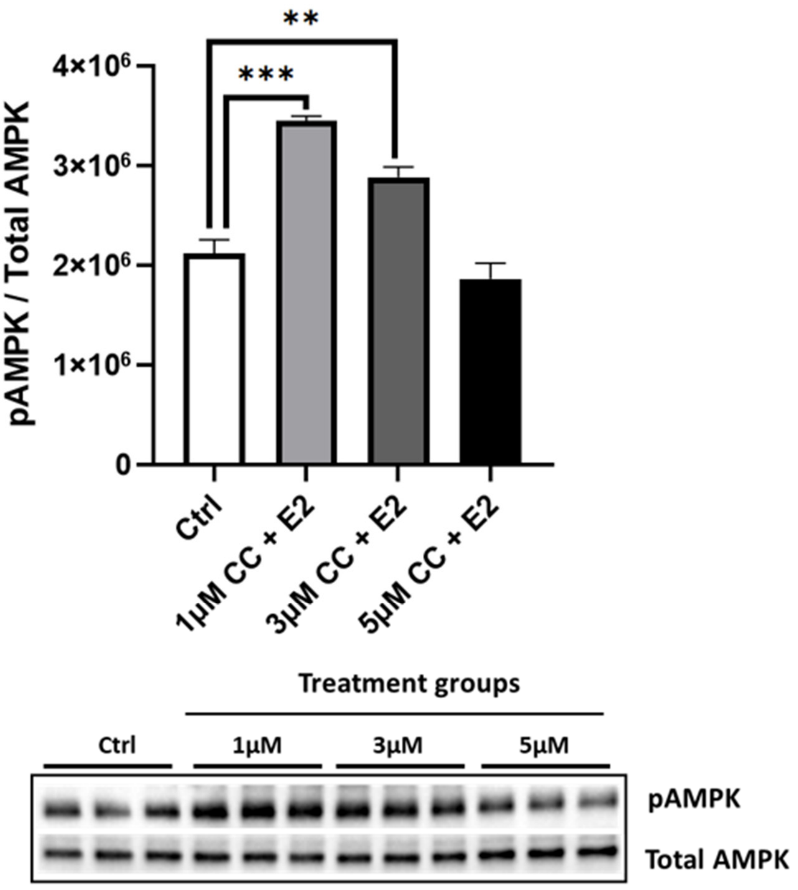

Supplementary Figure S1. Dose optimization of Compound C (CC) for AMPK inhibition in E2-treated cortical neurons. Primary cortical neurons were pretreated with increasing doses of CC (1 µM, 3 µM, and 5 µM) for 1 hour, followed by 10 nM E2 for 48 hours. Cell lysates were analyzed by Western blotting for pAMPK levels (normalized to total AMPK). Data are presented as mean ± SEM, with n=3 replicate cultures. Statistical significance was determined by one-way ANOVA followed by Dunnett's post hoc test; **p<0.01 and ***p<0.001. The 5 µM CC dose was selected for subsequent experiments due to its efficacy in blocking E2-induced AMPK phosphorylation.

Supplementary Figure S2.

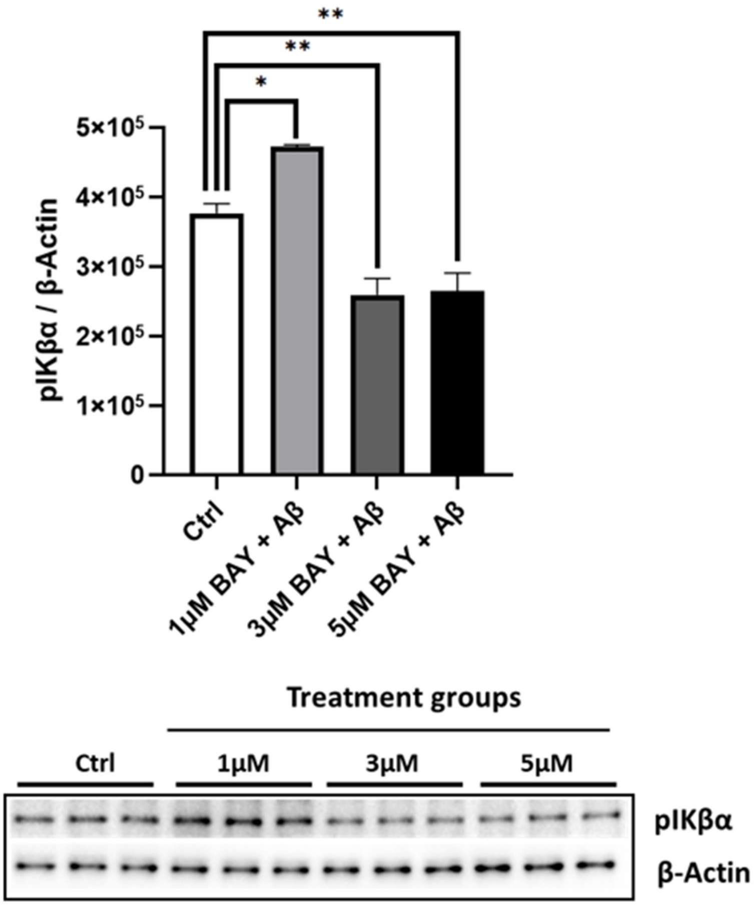

Supplementary Figure S2. Dose optimization of the NF-κB inhibitor BAY 11-7082 in Aβ-treated cortical neurons. Primary cortical neurons were pretreated with increasing doses of BAY 11-7082 (1 μM, 3 μM, and 5 μM) for 1 hour, followed by 10 μM Aβ exposure for 48 hours. Cell lysates were analyzed by Western blotting for phosphorylated IκBα (pIκBα) levels (normalized to β-actin). Data are presented as mean ± SEM, with n=3 replicate cultures. Statistical significance was determined by one-way ANOVA followed by Dunnett's post hoc test; *p<0.05 and **p<0.01. The 3 μM dose of BAY 11-7082 was selected for subsequent experiments due to its efficacy in suppressing Aβ-induced IκBα phosphorylation, indicating optimal NF-κB inhibition.
